# Tanshinone IIA attenuates bleomycin-induced pulmonary fibrosis in rats

**DOI:** 10.3892/mmr.2015.3333

**Published:** 2015-02-11

**Authors:** HUANYU HE, HAIYING TANG, LILI GAO, YUN WU, ZHIQIANG FENG, HONGLI LIN, TAIHUA WU

**Affiliations:** 1 Department of Respiratory Medicine, The First Affiliated Hospital of Dalian Medical University, Dalian, Liaoning 116011, P.R. China; 2 Department of Nephrology, The First Affiliated Hospital of Dalian Medical University, Dalian, Liaoning 116011, P.R. China

**Keywords:** idiopathic pulmonary fibrosis, bleomycin, tanshinone IIA, inflammation, fibrosis

## Abstract

Idiopathic pulmonary fibrosis is a chronic and progressive fibrotic lung disorder with unknown etiology and a high mortality rate. Tanshinone IIA (Tan IIA) is a lipophilic diterpene extracted from the Chinese herb *Salvia miltiorrhiza* Bunge with diverse biological functions. The present study was conducted to evaluate the effects of Tan IIA on bleomycin (BLM)-induced pulmonary fibrosis in rats. Rats received an intraperitoneal injection of Tan IIA and normal rats were used as controls. Severe pulmonary edema, inflammation and fibrosis were observed in the BLM-treated rats and the counts of total cells, neutrophils and lymphocytes were significantly increased in the bronchoalveolar lavage fluids of those rats. These pathological changes were markedly attenuated by subsequent treatment with Tan IIA. In addition, BLM-induced increased expression of tumor necrosis factor-α, interleukin (IL)-1β, IL-6, cyclooxygenase-2, prostaglandin E2, malondialdehyde, inducible nitric oxide synthase and nitric oxide in rats, which was also suppressed by Tan IIA injection. The present findings suggest therapeutic potential of Tan IIA for pulmonary fibrosis.

## Introduction

Idiopathic pulmonary fibrosis (IPF) is the most common fibrotic lung disorder with a poor prognosis and high mortality rate (1–2). Its pathogenesis is hypothesized to involve excessive fibroblast proliferation, abnormal reepithelialization and dysregulated extracellular matrix accumulation following alveolar injury, leading to progressive respiratory failure (3). Although the precise etiology of IPF remains to be elucidated, numerous previous studies have indicated that IPF may result from an interstitial inflammatory response to an unknown infection or injury (4). In addition, markers of oxidative stress have been identified in patient lungs with IPF and aberrant antioxidant activity has been demonstrated to exacerbate pulmonary fibrosis in several animal models (5). Additionally, nitric oxide (NO) has been observed to lead to acute inflammation in the lung (6). These earlier results suggest that reactive oxygen species (ROS) and reactive nitrogen species also have important roles in this disease. Although an antifibrotic drug, pirfenidone, has been approved for the treatment of IPF in Japan (7) and Europe (8), searching for novel effective therapeutic drugs for IPF is still required.

Tanshinone IIA (Tan IIA) is an important lipophilic diterpene extracted from the root of a Chinese herb termed *Salvia miltiorrhiza* Bunge (9). Ample evidence has demonstrated that Tan IIA exerts antitumoral effects during tumorigenesis of numerous types of cancer, including prostate cancer (10), colon carcinoma (11) and breast cancer (12). Tan IIA is also used as an effective remedy for the treatment of cardiovascular (13,14) and cerebrovascular (15) diseases. Furthermore, a study by Chen *et al* (15) has revealed that Tan IIA effectively inhibits the release of proinflammatory cytokines, including tumor necrosis factor-α (TNF-α) and interleukin (IL)-6, in a rat model of cerebral ischemia/reperfusion injury (15), suggesting that Tan IIA possesses anti-inflammatory potential. Notably, several previous lines of evidence have demonstrated a therapeutic effect of Tan IIA on experimental liver fibrosis in rodent models (16,17). In addition, similar inhibitory effects of Tan IIA on peritoneal fibrosis have also been reported in a peritoneal dialysis rat model (18). These previous findings suggest a protective role of Tan IIA in fibrotic diseases. However, there is a lack of data regarding the effects of Tan IIA on fibrotic lung disease.

Therefore, the present study was conducted to investigate the effects of Tan IIA on IPF in a bleomycin (BLM)-induced pulmonary fibrosis rat model. The underlying regulatory mechanisms associated with the potential anti-inflammatory and antifibrotic effects of Tan IIA were also investigated.

## Materials and methods

### Animals and treatment

Sprague-Dawley rats (age, 8 weeks; weight, ~250 g) were obtained from Dalian Medical University (Dalian, China), and maintained at a constant room temperature (20–22˚C) and humidity (50–60%) with a 12 h light/dark cycle, and with access to a standard diet and tap water *ad libitum*. All animal procedures were approved by the institutional animal care and use committee of Dalian Medical University, which conforms to the provisions of the Declaration of Helsinki in 1995 (as revised in Edinburgh 2000).

According to previously described methods (19,20), a pulmonary fibrosis rat model was reproduced in the present study. Briefly, rats were anesthetized with an intraperitoneal injection of 10% chloride hydrate (3.5 ml/kg body weight; Sinopharm Chemical Reagent Beijing Co., Ltd, Beijing, China) and then administered a single intratracheal instillation of BLM dissolved in saline (5 mg/kg body weight; Melone Pharmaceutical Co., Ltd, Dalian, China). Control rats received an equal volume of sterile saline using the same procedure. To examine the role of Tan IIA in BLM-induced pulmonary fibrosis pathogenesis, control and BLM-treated rats were administered daily intraperitoneal injections of Tan IIA(15 mg/kg body weight; Melone Pharmaceutical Co., Ltd) following BLM infusion. Accordingly, the rats were divided into four groups (n=25 per group): i) Control group; ii) Tan IIA group, control rats receiving a daily Tan IIA injection; iii) BLM; iv) BLM+Tan IIA, BLM-treated rats receiving a daily Tan IIA injection. On day 28 after the initial instillation rats were sacrificed by intraperintoneal injection of 10% chloral hydrate (5 ml/kg body weight).

### Lung index assay

Rats were anesthetized and sacrificed on day 28, and their lungs were removed and weighed prior to and following drying in an incubator at 60˚C for 72 h. The pulmonary edema was calculated as a wet/dry (W/D) weight ratio.

### Bronchoalveolar lavage (BAL)

BAL was performed three times by intratracheal instillation of 1.5 ml sterile saline in the trachea. The BAL fluids (BALF) were immediately centrifuged at 500 × g at 4˚C for 15 min. Cell-free supernatant of the first BAL sample was used to measure the secretion levels of cytokines (TNF-α, IL-1β and IL-6) using an Enzyme-Linked Immunosorbent Assay (ELISA) kit (USCN Life Science, Wuhan, China) according to the manufacturer’s instructions. The cytokine contents were expressed as pg/ml of BALF. The cell pellet was resuspended in cold phosphate-buffered saline (PBS; HyClone, Logan, UT, USA) and then subjected to assessment for total cell counts. To determine the differential cell counts, 200 cells from each BALF sample were stained with a Wright-Giemsa Stain kit (Jiancheng Bioengineering Institute, Nanjing, China), counted under a microscope (DP73; Olympus, Tokyo, Japan) and expressed as a percentage of total cells.

### Histological examination

Following sacrifice, the right lungs were removed and fixed in 4% paraformaldehyde, dehydrated, and embedded in paraffin. The paraffin-embedded tissue samples were sectioned into 5-*μ*m slices and then stained with hematoxylin & eosin (H&E) and Masson’s trichrome, and examined under a light microscope (DP73; Olympus). The Ashcroft score was used to determine the degree of fibrosis in lung specimens (21,22).

### Immunohistochemical staining

For immunohistochemical staining analysis, the 5-*μ*m slices were deparaffinized in xylene, hydrated with graded alcohol and then washed with 1% PBS. Subsequently, these slices were first incubated in 10 mmol/l citrate buffer (pH 6.0) at 100˚C for 10 min to retrieve the antigen and then placed in 3% hydrogen peroxide for 15 min at room temperature to block the endogenous peroxidase activity. Following rinsing with 1% PBS three times, the sections were blocked with goat serum for 30 min, incubated with rabbit polyclonal primary antibodies against the cyclooxygenase-2 (COX-2; 1:100 diluted; Wanlei Life Sciences, Shenyang, China) and inducible nitric oxide synthase (iNOS; 1:100 diluted; Wanlei Life Sciences) at 4˚C overnight. Subsequently, the sections were washed with 1% PBS, incubated with the corresponding goat anti-rabbit biotin-labeled secondary antibody (1:200; Beyotime, Shanghai, China) at 37˚C for 30 min and subsequently incubated in avidin-horseradish peroxidase complex (Beyotime). Thereafter, the sections were visualized using 3,3′-diaminobenzidine (Solarbio, Beijing, China) and counterstained with hematoxylin (Solarbio).

### Western blot analysis

Protein extracts from rat lung tissues were prepared using ice-cold NP-40 lysis buffer (Beyotime). Following determination of the protein concentration with bicinchoninic acid protein assay kit (Beyotime), equal quantities of protein samples were fractionated on SDS-PAGE, transferred onto polyvinylidene difluoride membranes (Millipore, Bedford, MA, USA), and blocked with 5% (w/v) skimmed milk (Yili Industrial Group Company, Hohhot, China). The membranes were blotted with mouse polyclonal antibodies against COX-2 (1:500 diluted; Wanlei Life Sciences) and iNOS (1:500 diluted; Wanlei Life Sciences) at 4˚C overnight and then incubated with the corresponding goat anti-rabbit horseradish peroxidase-conjugated secondary antibodies (1: 5,000 diluted; Beyotime) at room temperature for 45 min. The protein blots on the membranes were visualized using an ECL kit (Millipore), and the band density values were calculated as a ratio to β-actin.

### Measurements of NO, prostaglandin E2 (PGE2) and malondialdehyde (MDA) levels in lung tissue samples

NO is usually oxidized to nitrate and nitrite *in vivo* (23), therefore the total NO levels were determined by assessing the sum of nitrate and nitrite based on the Griess reaction using a total NO assay kit (Beyotime). A standard curve was established with a set of serial dilutions of sodium nitrite. NO production levels were expressed as μmol/mg protein. In addition, the pulmonary protein expression of prostaglandin E2 (PGE2) was determined using an ELISA, and expressed as pg/mg protein. MDA content in rat lung tissue samples was detected using an MDA assay kit (Jiancheng Bioengineering Institute, Nanjing, China), and expressed in nmol/mg protein.

### Statistical analysis

The present data were expressed as the mean ± standard deviation. Statistical differences among several groups were determined using a one-way analysis of variance followed by the Bonferroni post hoc test using SPSS version 17.0 (SPSS, Chicago, IL, USA). P<0.05 was considered to indicate a statistically significant difference.

## Results

### Tan IIA attenuates the inflammatory responses in BLM-induced pulmonary fibrosis

To determine the effect of Tan IIA on BLM-induced pulmonary inflammation responses in rats, the inflammatory cell counts in BALF were initially assayed. As indicated in Table I, a significant influx of inflammatory cells was observed in BALF of the rats with pulmonary fibrosis, and the percentages of neutrophils and lymphocytes were also markedly increased. Following the Tan IIA injection, BLM-induced increases of inflammatory cell counts were markedly inhibited.

**Figure 1:**
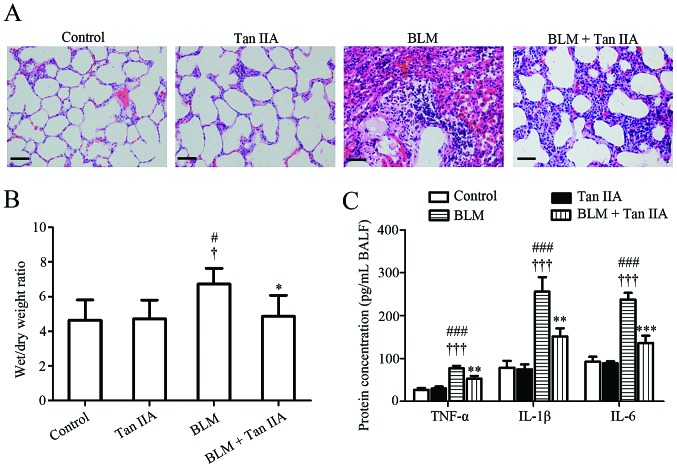
Effects of Tan IIA on histological changes of rat lung tissues. (A) Representative images of hematoxylin & eosin staining, scale bars=50 *μ*m. (B) Wet/dry weight ratio of rat lung (n=6 per group). (C) Secretion levels of TNF-α, IL-1β and IL-6 in BALF quantified by ELISA (n=3 per group). Results are expressed as the mean ± standard deviation. ^#^P<0.05 and ^###^P<0.001, versus the control group; ^†^P<0.05 and ^†††^P<0.001, versus the Tan IIA group; *P<0.05, ^**^P<0.01 and ^**^*P<0.001, versus the BLM group. TNF-α, tumor necrosis factor-α; IL-1β, interleukin 1β; IL-6, interleukin-6; BALF, bronchoalveolar lavage fluids; Tan IIA, Tanshinone IIA; BLM, bleomycin.

H&E staining results revealed that, compared with lung tissues obtained from control or Tan IIA groups, the lung structure of rats from the BLM-treated group was severely damaged and the lung interstitium was evidently thickened (Fig. 1A). These pathological changes were attenuated by the intraperitoneal injection of Tan IIA. Since the thickening of the lung interstitium may due to extra fluid (edema) or inflammation (24), the lung W/D ratio and the secretion levels of the pro-inflammatory factors in the experimental rats were subsequently detected. It was noted that BLM-induced pulmonary edema and elevation of TNF-α, IL-1β and IL-6 in BALF were alleviated by Tan IIA injection (Fig. 1B and C). Collectively, these results suggested that Tan IIA may protect the lung against the deleterious inflammation induced by BLM.

### Tan IIA mitigates BLM-induced pulmonary fibrosis in rats

**Figure 2:**
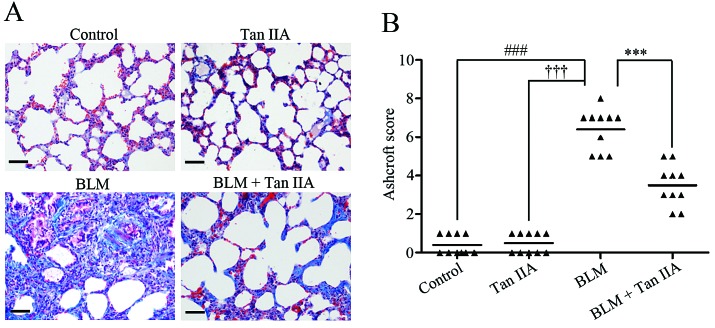
Tan IIA attenuates BLM-induced pulmonary fibrosis in rats. (A) Representative images of Masson’s trichrome staining, scale bars=50 *μ*m. (B) Comparison of the Ashcroft score among the experimental groups (n=10 per group). Results are expressed as the mean ± standard deviation. ^###^P<0.001, versus the control group; ^†††^P<0.001, versus the Tan IIA group; ***P<0.001, versus the BLM group. Tan IIA, Tanshinone IIA; BLM, bleomycin.

Masson’s trichrome staining results revealed severe pulmonary fibrosis in BLM-treated rats (Fig. 2A). Following injection of Tan IIA, the area of collagen deposition was markedly reduced in rats treated with BLM (Fig. 2A). The Ashcroft score was then used to determine the extent of lung fibrosis. The data indicated that, following Tan IIA administration, the score of rats with pulmonary fibrosis was significantly decreased (Fig. 2B). These findings indicated an antifibrotic effect of Tan IIA on pulmonary fibrosis in rats.

### Tan IIA attenuates oxidative stress in BLM-induced pulmonary fibrosis in rats

**Figure 3:**
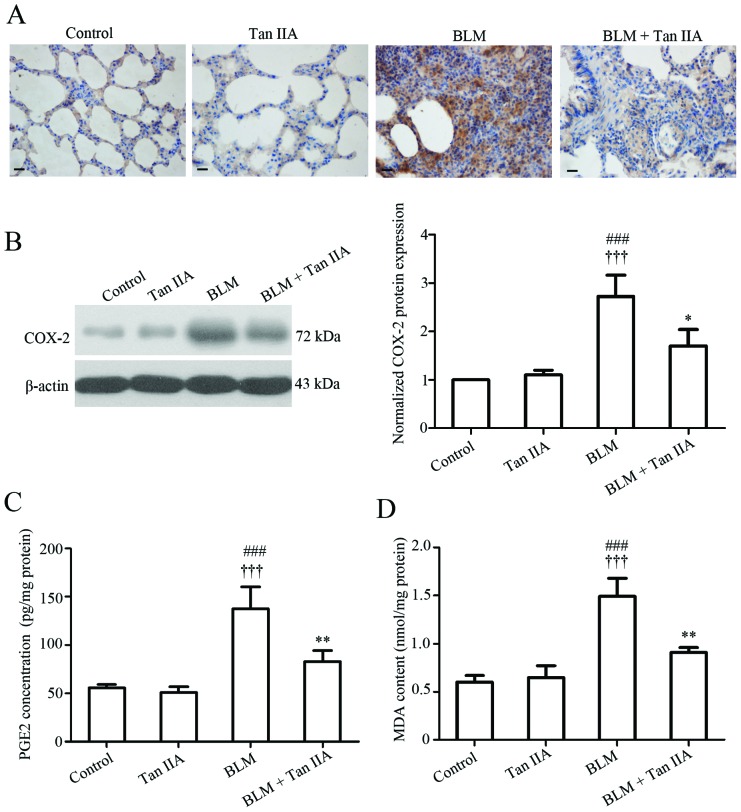
Effects of Tan IIA on oxidative stress-associated factors in lung tissue. (A) Representative images of immunohistochemical staining for COX-2 protein in lung tissue samples, scale bars=20 *μ*m. (B) COX-2 protein expression in lung tissue specimens determined by western blot analysis. (C) PGE2 expression and (D) MDA concentration in lung tissues. Results are expressed as the mean ± standard deviation, n=3 per group. ^###^P<0.001, versus the control group; ^†††^P<0.001, versus the Tan IIA group; *P<0.05 and ***P<0.001 versus the BLM group. COX-2, cyclooxygenase-2, PGE2, prostaglandin E2; MDA, malondialdehyde; Tan IIA, Tanshinone IIA; BLM, bleomycin.

Numerous studies have observed that oxidative stress is involved in the fibrotic processes of various organs, including the lung (25). Since reduction-oxidation products are difficult to measure directly in tissues, the effect of Tan IIA on oxidative stress was evaluated by detecting the expression of several key factors implicated in this reaction, including COX-2, PGE2 and MDA. Data from immunohistochemistry (Fig. 3A) and western blot analyses (Fig. 3B) indicated that pulmonary COX-2 protein expression increased following BLM treatment, but decreased by the following Tan -A injection. In addition, the ELISA results revealed that the expression pattern of COX-2 enzymatic product, PGE2 (26) paralleled that of COX-2 in rat lungs (Fig. 3C). Additionally, BLM-induced elevated expression of the final product of polyunsaturated fatty acid peroxidation, MDA (27), was also inhibited by Tan -A administration (Fig. 3D). These results revealed that Tan IIA may suppress the abnormal oxidative reaction caused by BLM in rat lungs.

### Tan IIA inhibits iNOS expression and NO production in BLM-induced pulmonary fibrosis

**Figure 4:**
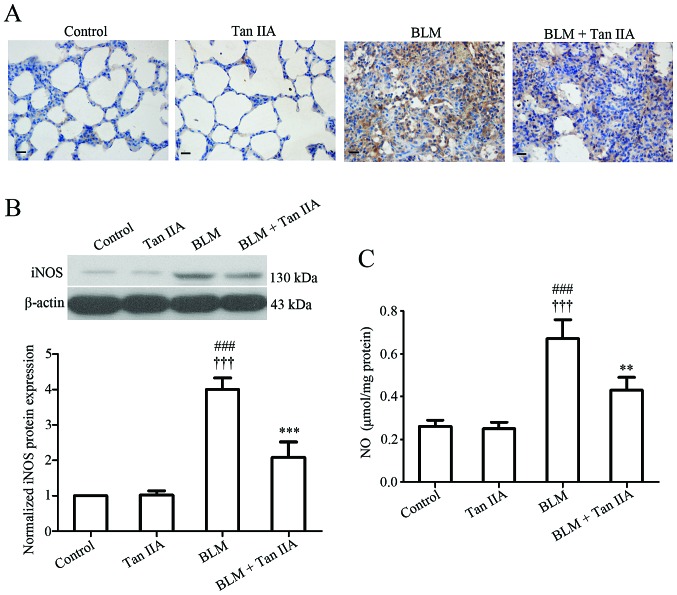
Tan IIA reduces BLM-induced upregulation in pulmonary iNOS expression and NO production in rats. (A) Representative images of immunohistochemical staining for iNOS protein in lung tissue samples, scale bars=20 *μ*m. (B) iNOS protein expression in lung tissue specimens determined by western blot analysis. (C) Total NO levels, the sum of nitrate and nitrite in lung tissues quantified using Griess reaction method. Results are expressed as the mean ± standard deviation, n=3 per group. ^###^P<0.001 versus the control group; ^†††^P<0.001 versus the Tan IIA group; **P<0.01, ***P<0.001 versus the BLM group. iNOS, inducible nitric oxide synthase; NO, nitric oxide. Tan IIA, Tanshinone IIA; BLM, bleomycin.

Regarding the potent implication of iNOS-derived NO in the pathogenesis of human pulmonary fibrosis (28), iNOS protein expression and NO production level were measured in rat lung tissue samples. Immunodetection (Fig. 4A) and western blot analysis (Fig. 4B) indicated that Tan IIA injection induced a significant reduction of iNOS expression in rats treated with BLM. In addition, the ELISA results revealed that the pulmonary NO level was increased following BLM instillation, but decreased after Tan IIA treatment (Fig. 4C). The present data suggested that Tan IIA injection may reduce BLM-induced excessive NO production in rat lungs.

## Discussion

BLM-induced pulmonary fibrosis is a well-established disease model for IPF and widely used in the investigation of the efficacy and mechanism of therapeutic candidates (29,30). In the present study, pulmonary fibrosis was induced in rats by one-off instillation of BLM, and the potential effects of Tan IIA on the BLM-induced fibrotic lesions of rat lungs were determined. The present data indicated that administration of Tan IIA reduced BLM-induced inflammatory cell infiltration, pro-inflammatory cytokine release and excessive pulmonary collagen deposition in rat lung tissues. In addition, COX-2-associated oxidative reaction and iNOS-derived NO production in the BLM treated rats were also inhibited by Tan IIA injection.

Following BLM administration, increased numbers of inflammatory cells were observed in the BALF of rats. In addition, the secretion of TNF-α, IL-1β and IL-6 in rat lung tissue samples was enhanced following BLM treatment. Pulmonary edema and fibrosis were also detected in BLM-treated rats. The aforementioned observations confirmed the validity of the BLM-induced pulmonary fibrotic lesion model. These pathological alterations induced by BLM were significantly attenuated by Tan IIA injection. Although similar anti-inflammatory and antifibrotic effects of Tan IIA have been reported in previous studies (17,31–33), to the best of our knowledge, this is the first study demonstrating a therapeutic effect of Tan IIA in pulmonary fibrosis.

The lungs are constantly exposed to relatively higher oxygen tensions than other organs. The exogenous oxidants may increase oxidant production and activate inflammatory cells to generate free radicals in the lungs (34). Oxidative stress is an imbalance between the generation of ROS and the capacity to detoxify these intermediates (35), and it has a major role in the pathogenesis of pulmonary fibrosis (25). A previous study demonstrated that Tan IIA inhibits angiotensin II-induced ROS formation in rat cardiac fibroblasts (36). Additionally, Tan IIA has also been demonstrated to exert protective effects in rat kidneys by attenuating oxidative stress injury (37). However, by contrast, a study by Chiu and Su (38) has indicated that Tan IIA promotes the production of ROS in human lung cancer cells (38). These earlier studies suggest inconsistent effects of Tan IIA on oxidative reactions. To address this issue, the expression levels of certain key factors involved in oxidative stress were examined. It was noted that BLM-induced increased expression of redox-responsive COX-2 and its enzymatic product PGE2 in rat lungs was significantly inhibited by Tan IIA injection. Additionally, BLM-induced elevation of the oxidative stress biomarker, MDA (39), in rat lungs were also reduced following Tan IIA treatment. The present findings revealed an anti-oxidative effect of Tan IIA in pulmonary fibrosis, which was supported by several previously reported studies (40–42). However, the exact mechanisms underlying the regulatory role of Tan IIA in the oxidative reaction in pulmonary fibrosis require further investigation.

In addition to ROS, an overproduction of NO also has an important role in various disease models, including BLM-induced fibrotic lung disease (43). An earlier study demonstrated that inhibition or knockout of iNOS results in resistance to BLM-induced lesions in mice (44). Based on these results, it was hypothesized that Tan IIA may inhibit iNOS expression and the subsequent NO production in BLM-induced pulmonary fibrosis. The results confirmed this hypothesis by demonstrating a significant reduction of pulmonary iNOS expression and NO production levels in BLM-treated rats following Tan IIA injection, corresponding to previous lines of evidence (32,45). In addition, considering evidence that the iNOS-derived NO affects the lung inflammatory response in mice (46), Tan IIA may exert its anti-inflammatory effect by modulating NO production during the development of pulmonary fibrosis.

In conclusion, the present study demonstrates that administration of Tan IIA reduces BLM-induced inflammatory cell infiltration, pro-inflammatory cytokine release and excessive collagen deposition in the rat lung. Additionally, abnormal oxidative reactions and NO production in the BLM-treated rats are inhibited by Tan IIA administration. The present findings therefore suggest a potential protective effect of Tan IIA against pulmonary fibrosis.

## Figures and Tables

**Table I:** Effect of Tan IIA on BLM-induced changes in total and differential cell counts in the bronchoalveolar lavage fluid of rats.

Group	Total cells (×10^5^/ml)	Neutrophils (%)	Lymphocytes (%)	Macrophages (%)
Control	1.62±0.30	8.42±2.15	13.00±3.65	78.58±5.53
Tan IIA	1.76±0.29	8.75±2.70	11.67±4.07	79.58±6.70
BLM	12.10±1.62^c^^d^	24.17±2.88^c^^d^	25.58±3.77^c^^d^	50.25±5.99^c^^d^
BLM+Tan IIA	7.41±0.83^b^	18.92±3.06^a^	19.33±2.71^a^	61.75±5.35^a^

Data are expressed as the mean ± standard deviation (n=6 per group);

a P<0.05 and

b P<0.001 compared with the BLM group;

c P<0.001 compared with control group;

d P<0.001 compared with Tan IIA group. Tan IIA, Tanshinone IIA; BLM, bleomycin.
